# Modeling of *ACTN4*-Based Podocytopathy Using *Drosophila* Nephrocytes

**DOI:** 10.1016/j.ekir.2022.10.024

**Published:** 2022-10-31

**Authors:** Johanna Odenthal, Sebastian Dittrich, Vivian Ludwig, Tim Merz, Katrin Reitmeier, Björn Reusch, Martin Höhne, Zülfü C. Cosgun, Maximilian Hohenadel, Jovana Putnik, Heike Göbel, Markus M. Rinschen, Janine Altmüller, Sybille Koehler, Bernhard Schermer, Thomas Benzing, Bodo B. Beck, Paul T. Brinkkötter, Sandra Habbig, Malte P. Bartram

**Affiliations:** 1 Department II of Internal Medicine and Center for Molecular Medicine Cologne, Faculty of Medicine, University of Cologne, University Hospital Cologne, Cologne, Germany; 2 Cologne Excellence Cluster on Cellular Stress Responses in Aging-Associated Diseases, Faculty of Medicine, University of Cologne, University Hospital Cologne, Cologne, Germany; 3 Center for Molecular Medicine Cologne, Faculty of Medicine, University of Cologne, University Hospital Cologne, Cologne, Germany; 4 Institute of Human Genetics, Faculty of Medicine, University of Cologne, University Hospital Cologne, Cologne, Germany; 5 Department of Pediatrics, Faculty of Medicine, University of Cologne, University Hospital Cologne, Cologne, Germany; 6 Department of Pediatrics, Division of Pediatric Nephrology, University of Bonn, Bonn, Germany; 7 Mother and Child Health Care Institute of Serbia “Dr Vukan Čupić,” Department of Nephrology, Faculty of Medicine, University of Belgrade, Belgrade, Serbia; 8 Institute of Pathology, University Hospital of Cologne, Cologne, Germany; 9 Department of Biomedicine, Aarhus University, Aarhus, Denmark; 10 Aarhus Institute of Advanced Studies, Aarhus University, Aarhus, Denmark; 11 III Medical Clinic, University Hospital Hamburg Eppendorf, Hamburg, Germany; 12 Berlin Institute of Health at Charité-Universitätsmedizin Berlin, Max Delbrück Center for Molecular Medicine, Berlin, Germany; 13 Cologne Center for Genomics, University of Cologne, Cologne, Germany

**Keywords:** *ACTN4*, *Drosophila*, FSGS, nephrocyte, nephrotic syndrome, podocyte

## Abstract

**Introduction:**

Genetic disorders are among the most prevalent causes leading to progressive glomerular disease and, ultimately, end-stage renal disease (ESRD) in children and adolescents. Identification of underlying genetic causes is indispensable for targeted treatment strategies and counseling of affected patients and their families.

**Methods:**

Here, we report on a boy who presented at 4 years of age with proteinuria and biopsy-proven focal segmental glomerulosclerosis (FSGS) that was temporarily responsive to treatment with ciclosporin A. Molecular genetic testing identified a novel mutation in alpha-actinin-4 (p.M240T). We describe a feasible and efficient experimental approach to test its pathogenicity by combining *in silico*, *in vitro*, and *in vivo* analyses.

**Results:**

The *de novo* p.M240T mutation led to decreased alpha-actinin-4 stability as well as protein mislocalization and actin cytoskeleton rearrangements. Transgenic expression of wild-type human alpha-actinin-4 in *Drosophila melanogaster* nephrocytes was able to ameliorate phenotypes associated with the knockdown of endogenous actinin. In contrast, p.M240T, as well as other established disease variants p.W59R and p.K255E, failed to rescue these phenotypes, underlining the pathogenicity of the novel alpha-actinin-4 variant.

**Conclusion:**

Our data highlight that the newly identified alpha-actinin-4 mutation indeed encodes for a disease-causing variant of the protein and promote the *Drosophila* model as a simple and convenient tool to study monogenic kidney disease *in vivo*.

## Graphical abstract

**Figure:**
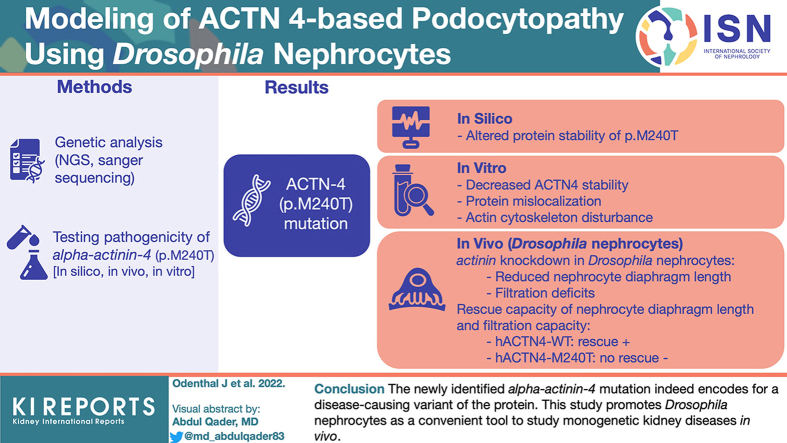

FSGS represents a histopathological pattern of injury and is classified as primary or secondary depending on whether an underlying cause can be identified. These entities are further subdivided on the basis of their response to immunosuppressive therapy, relapse frequency, and histologic subvariants and whether genetic variants can be identified. Clinically, FSGS can present with proteinuria in the nephrotic or subnephrotic range, and signs of nephrotic syndrome may be present.^1^ The prevalence of FSGS is rising, and FSGS is a major contributor to ESRD requiring dialysis or kidney transplantation.^2^^,^^3^ The driving factors leading to FSGS are diverse and differ between pediatric and adult populations. Although immunologic causes and genetic mutations predominate in children, obesity or reduced nephron mass leading to glomerular hyperfiltration, viral infections, or autoimmune diseases as well as medications are further factors in adult populations. Therefore, a very careful examination of each patient is required to (i) determine the underlying cause, (ii) optimize the patient’s treatment and counseling, and (iii) predict the risk of recurrence after kidney transplantation.

A monogenetic form of glomerular and podocyte disorders can be detected in up to one-third of patients.^4^ Genotype information is important for personalized treatment approaches because it predicts response to treatment options and risk of FSGS recurrence after transplantation, helps to avoid unnecessary drug toxicity and side effects, and aids in the selection of an appropriate kidney donor in the case of a living transplant strategy as well as the assessment of other family members at risk. To date, more than 50 FSGS-associated genes have been discovered, most of which encode proteins that play central roles in regulating the podocyte’s architecture and function.^5^ Among these, especially proteins associated with the slit diaphragm such as nephrin or podocin and components of the cytoskeleton and cytoskeleton-associated proteins such as *ACTN4* and *INF2* were identified as causative for inherited forms of FSGS.^6^ Although most mutations in children are inherited in an autosomal recessive mode, there are autosomal-dominant forms with rather slowly progressive courses that present mainly in adults.

These patients often do not respond well to standard immunosuppressive therapy. However, recently, several case reports have been published reporting on partial or complete remission in particular types of genetic FSGS.^7^ Large cohort studies combining comprehensive analyses of both genotypes and phenotypes with a long-term follow-up over several years will be essential to stratify patients to a specific management course. Recently, we established a clinical research unit (CRU329–www.podocyte.org) and a clinical registry (FOrMe registry, ClinicalTrials.gov identifier: NCT03949972^8^) in Germany. Within this registry, we provide comprehensive genetic analysis, monitor the clinical outcome, and provide *in vitro* and *in vivo* analysis of novel mutations to predict the outcome.

Here, we report on 3 patients with *de novo ACTN4* mutations that were identified within the clinical research unit and that developed kidney disease with a rapid deterioration of kidney function at a rather younger age than most published patients with mutations in *ACTN4*.^9^ The mutation of 1 patient was previously analyzed *in vitro*^10^. Applying *in silico*, *in vitro*, and *in vivo* analyses using the *Drosophila melanogaster* nephrocyte model, we now characterized the biological consequences of a further *ACTN4* mutation resulting in amino acid change p.M240T and demonstrate its pathogenicity. We established a thorough workflow to elucidate and evaluate the pathogenic potential of unknown variants that might be disease-causing in nephrotic syndrome, eventually guiding and facilitating further treatment options for the individual patient and hereby emphasize the genetic power of the *Drosophila* model in translational kidney research.

## Methods

### Probands

All investigations were conducted in accordance with the principles of the Declaration of Helsinki and after obtaining written informed consent from the patient and his parents. Clinical and biochemical data were collected from medical charts. Standard methods were used to analyze electrolytes, creatinine, and other laboratory parameters. This study was carried out with the approval of the ethics committee of the University Hospital Cologne (number 15-215).

### Genetic Analysis

DNA of patients 1 and 2 was analyzed by next-generation sequencing as described previously.^10^ We performed Sanger sequencing of DNA from patient 3 for 8 genes commonly found in mainly nonsyndromic steroid-resistant nephrotic syndrome (SRNS)/FSGS (*NPHS1*, *NPHS2*, *WT1*, *TRPC6*, *INF2*, *ACTN4*, *PLCE1*, and *PAX2*). Segregation of all parents DNA confirmed that all pathogenic *ACTN4* variants occurred *de novo* in the patients (see Supplementary Table S1).

### Analysis of Thermodynamic Stability *In Silico*

Computational analyses were carried out as described before^10^: The respective protein data bank files were obtained from the PubMed structure (protein data bank: 2EYN [*ACTN1*], 1WKU [*ACTN3*], 2R0O [*ACTN4*]). Because there is no crystal structure for wild-type (WT) *ACTN4* available, we used the structure of *ACTN4* K255E, which shows no significant structural change in comparison with the WT protein.^11^ The files were uploaded to the respective web servers, and calculation of free energy (delta-delta-G) was performed using the default settings. The effect of an amino acid substitution (M to P for the respective residues) was calculated.^12^, ^13^, ^14^, ^15^, ^16^

### Actin Fractionation Assay

Differential centrifugation was performed to investigate the subcellular localization of *ACTN4* and its clustering with components of the actin cytoskeleton. For this, Flag-tagged hACTN4-WT or hACTN4-M240T was transfected into human embryonic kidney 293 T cells, which were harvested in ice-cold phosphate-buffered saline the next day. Cells were lysed in lysis buffer (20-mM Tris-HCl, pH 7.5, 50-mM NaCl, 50-mM NaF, 15-mM Na_4_P_2_O_7_, 2-mM Na_3_VO_4_, 1% Triton X-100, and protease inhibitor mix complete protease inhibitor mix [Roche]). After low-speed centrifugation of the whole-cell lysate at 14,000 × *g* for 15 minutes, large cytoskeletal structures such as actin bundles were pelleted into the Triton insoluble fraction. The supernatant (Triton soluble fraction) was further centrifugated at 100,000 × *g* for 30 minutes, after which fractions were separated into F-actin (pellet) and G-actin (supernatant). The fractions were then analyzed by western blot.

### Fly Lines and Husbandry

Flies were kept on standard medium and maintained at 25 °C. As a nephrocyte-specific driver, we used Sticks and Stones–GAL4 (sns-GAL4)^17^ combined with upstream activating sequence (UAS)-dcr2,^18^ to achieve higher knockdown efficiency. UAS-GFP-RNA interference (RNAi) (BDSC #41553) served as control, and UAS-*actn*-RNAi-1 (VDRC #7762) and UAS-*actn*-RNAi-2 (VDRC #110719) were used to silence the actinin gene. Strains transgenically expressing the human hemagglutinin (HA)-tagged alpha-actinin-4 variants were generated via phi31-mediated recombination into the attP1:(2R) 55C4 locus (GenetiVision) and recombined with VDRC #110719 to generate stable lines expressing both UAS-*actn*-RNAi2 and HA-*hACTN4*-variants.

### Immunofluorescence Stainings in Nephrocytes

Immunofluorescence stainings in nephrocytes were performed as described before.^19^ In brief, nephrocytes were dissected in phosphate-buffered saline followed by fixation in 4% formaldehyde for 20 minutes and methanol for 1 hour at room temperature. After 3 washing steps in wash buffer (phosphate-buffered saline, 0.3% Triton, 0.5% bovine serum albumin), primary antibodies were incubated in wash buffer overnight at 4 °C. Primary antibodies were as follows: mouse antipolychaetoid 2 (Developmental Studies Hybridoma Bank, 1:25), goat anti–horseradish peroxidase (Jackson ImmunoResearch #123-005-021, 1:200), and rabbit anti–HA (Sigma #H6908, 1:100). After 3 washing steps and 30 minutes blocking in 5% normal donkey serum, the tissue was incubated with secondary antibodies (donkey anti-rabbitCy3, Jackson ImmunoResearch #711-165-152, 1:250; donkey anti-goatAlexa488, Jackson ImmunoResearch #705-545-003, 1:250; goat anti-mouseAtto647N, Sigma #50185, 1:1000) for 1 hour at room temperature. Following 3 washing steps, the tissue was mounted in Vectashield mounting medium (Linaris, H-1200).

### Tracer Uptake Assay in Nephrocytes

Tracer uptake assays were performed using fluorescein isothiocyanate (FITC)–labeled bovine albumin (Sigma#A9771) as described earlier.^19^ Nephrocytes were dissected in HL3 medium (70-mM NaCl, 5-mM KCl, 5-mM CaCl_2_∗2H_2_O, 20-mM MgCl_2_∗6H_2_O, 10-mM NaHCO_3_, 5-mM trehalose, 115-mM sucrose, 5-mM HEPES) and incubated in 0.2 mg/ml FITC-albumin for 30 seconds at room temperature. After rinsing the tissue with HL3 medium several times, nephrocytes were fixed for 20 minutes in 4% formaldehyde and mounted in Vectashield mounting medium (Linaris, H-1200).

### Microscopy and Image Analysis

Cultured cells were imaged using an Axiovert 200 microscope (C-Apochromat 63x/1.22 W objective, Carl Zeiss MicroImaging, Jena, Germany) using Axiovision 4.8 (Carl Zeiss MicroImaging) for acquisition and subsequent image processing. *Drosophila* nephrocytes were imaged with a Leica TCS SP8 confocal microscope using a 20× air objective with a numerical aperture of 0.75 (PL APO 20x/0.75 DRY, Leica Microsystems). Images were analyzed using Fiji^20^, and the fluorescence intensity of the nephrocytes was quantified as described previously.^19^ For high-resolution stimulated emission depletion (STED) images, a Leica TCS SP8 gSTED 3× microscope (Leica Microsystems) equipped with a white light laser for excitation and hybrid detectors (HyDs) for time-gated detection was used. After acquisition with a 100× oil immersion objective with a numerical aperture of 1.4 (PL APO 100×/1.4 Oil STED, Leica Microsystems), the images were further processed using the Huygens Essential software (Scientific Volume Imaging) for deconvolution. Quantification of the nephrocyte diaphragm (ND) length was done using a previously published Fiji macro.^21^

Further Methods are provided in Supplementary Methods.

## Results

### Clinical Course and Genetic Findings

All patients presented as sporadic cases of childhood onset of nephrotic syndrome (Supplementary Table S1). Patient 1 presented with SRNS at the age of 17 years. Renal biopsy confirmed FSGS, and genetic analysis by whole-exome sequencing identified the previously reported recurrent *ACTN4* p.F153L mutation. Proteinuria could be reduced by ciclosporin A treatment and supportive medication.

Patient 2 presented with ESRD at the age of 13 years. The identified *de novo* mutation in *ACTN4* (p.G195D) was analyzed *in vitro* and published before.^10^ Initiation of dialysis was necessary. After living-related kidney transplantation, no recurrence of FSGS occurred in the follow-up.

**Figure 1:**
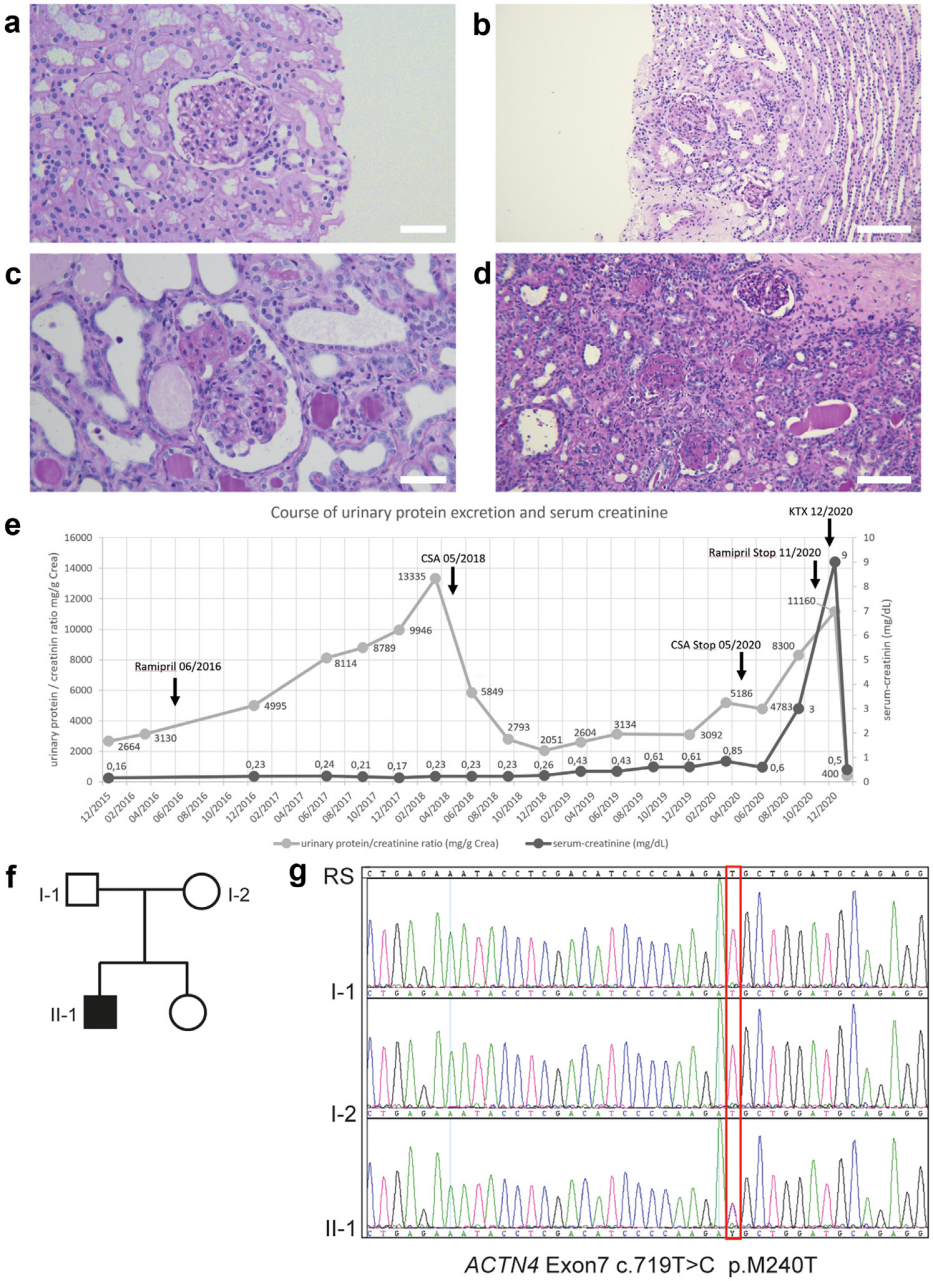
Identification of a novel *ACTN4* mutation in a patient presenting with steroid-resistant nephrotic syndrome. (a–d) Periodic acid–Schiff stainings of kidney biopsies of the index patient. (a) Initial kidney biopsy in 2015 showing glomeruli with normal appearing capillary tuft. Electron microscopy revealed foot process effacement of the podocytes leading to the diagnosis of minimal change glomerulopathy. (b) Kidney biopsy in 2017 with 1 glomerulum showing a perihilar focal and segmental glomerulosclerosis. (c,d) Nephrectomy specimen of (c) right and (d) left native kidney with increased globally sclerosed glomeruli and increased interstitial fibrosis and tubular atrophy. (e) Overview of the clinical course of the patient. Gray: urinary protein/creatinine ratio in mg/g. Black: serum-creatinine level in mg/dl. The patient first presented with proteinuria in December 2015. After a first biopsy (2015) showing minimal change glomerulopathy and, in the absence of clinical nephrotic syndrome, rampiril was initiated in June 2016. A second biopsy in June 2017 confirmed focal segmental glomerulosclerosis. Therapy with CSA resulted in a prompt but transient decrease of proteinuria and was stopped in May 2020 when renal function declined and proteinuria increased despite treatment. The patient was then prepared for kidney transplantation and received a living-donor graft in December 2020. (f,g) Pedigree of the index patient and targeted Sanger sequencing of *ACTN4* exon 7. Electropherograms depict the relevant sequence section around c.719T>C (p.M240T) in the index patient (II-1) and both parents (I-1 and I-2). Scale bars: 50 μm in (a) and (c), 100 μm in (b) and (d). CSA, ciclosporin A; KTx, kidney transplantation; RS, reference sequence.

Patient 3 presented with asymptomatic proteinuria at the age of 4 years. A first kidney biopsy at the age of 5 years showed minimal change glomerulopathy (Figure 1a). Comprehensive workup excluded immunologic or infectious causes. Because proteinuria persisted though treatment with angiotensin-converting enzyme inhibition (Figure 1e), a second kidney biopsy was performed at the age of 6 years that revealed FSGS (Figure 1b).

At the same time, a gene panel analysis of 8 genes commonly found in mainly nonsyndromic SRNS/FSGS (*NPHS1*, *NPHS2*, *WT1*, *TRPC6*, *INF2*, *ACTN4*, *PLCE1*, and *PAX2*) was performed and yielded unremarkable results except for a heterozygous variant c.719T>C (p.M240T) in *ACTN4*. Segregation of the parents’ DNA confirmed that p.M240T occurred *de novo* in the patient (Figure 1f and g). The identified ACTN4 variant p.M240T could not be found in either large genome databases (gnomAD^22^ and HGMD mutational databases [HGMD professional 2022.2]) or our local rare kidney disease database.

Therapy with ciclosporin A was initiated (targeted blood level trough 80–120 ng/ml) and resulted in a significant decrease of proteinuria (Figure 1e). In 2020, at the age of 9 years, however, the patient developed increasing proteinuria, edema, and deterioration of kidney function (Figure 1e). Ciclosporin A therapy was discontinued at that time, and the patient progressed to end-stage kidney failure within 6 months. He received a preemptive living-donor kidney transplant from his father and has had a stable transplant function since then. A nephrectomy of both native kidneys was performed (Figure 1c and d) due to persisting proteinuria, and since removal, the boy has not shown any signs of proteinuria.

### *ACTN4*-M240T Leads to Decreased Stability *In Silico* and *In Vitro*

The *ACTN4* mutation c.719T>C leads to amino acid change p.M240T in the N-terminal actin-binding calponin-homology domain. This is similar to other, previously described disease-associated mutations such as p.W59R, p.G195D, and p.K255E, which were reported to result in altered protein stability.^10^^,^^23^^,^^24^

**Figure 2:**
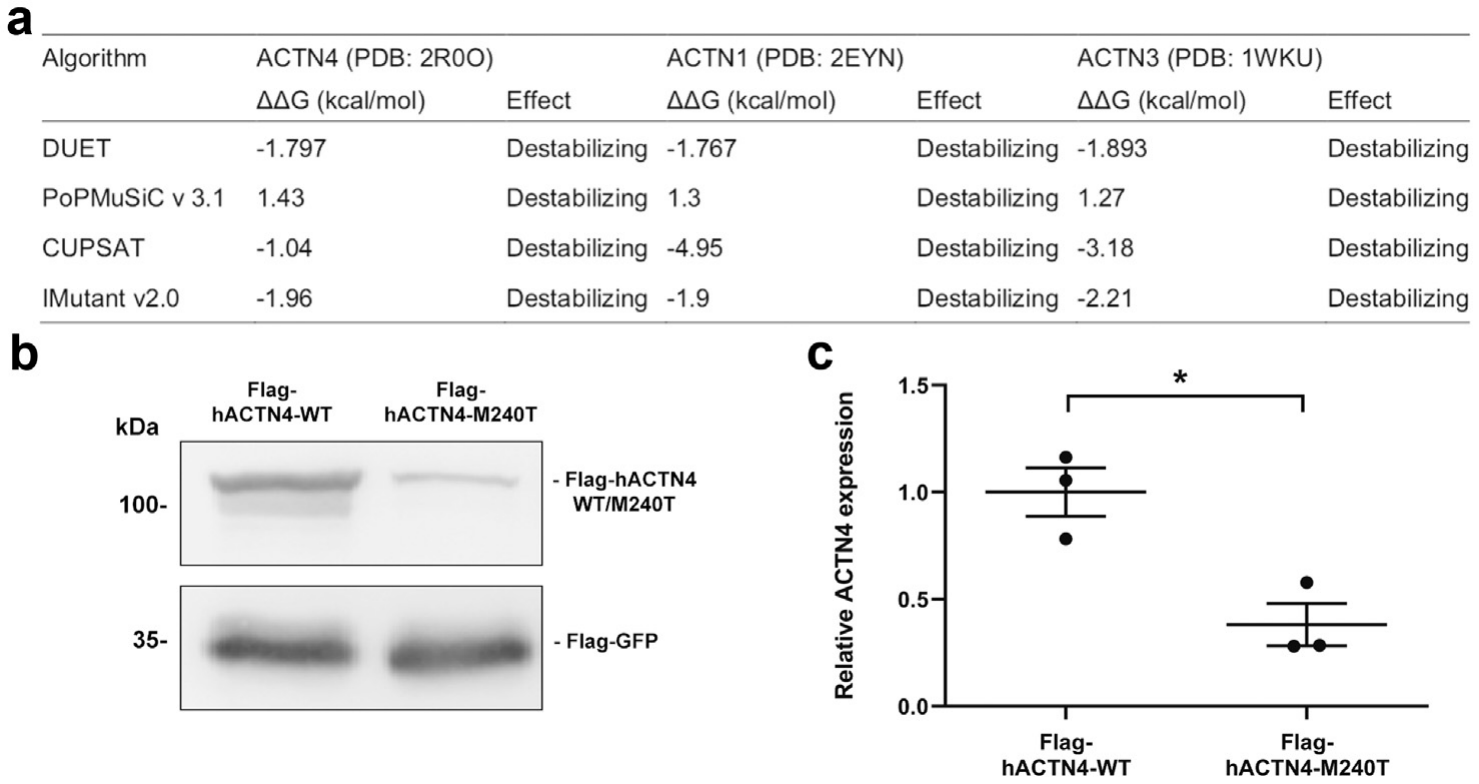
*In silico* and *in vitro* characterization of hACTN4-M240T stability. (a) *In silico* prediction of M240T mutation on alpha-actinin protein stability (delta-delta-G) using 4 different computational algorithms. M>T amino acid exchange in the conserved domain has a destabilizing effect in *ACTN4* as well as *ACTN1* and *ACTN3*. (b) Representative western blot analysis of whole-cell lysates cotransfected with Flag-hACTN4-WT or Flag-hACTN4-M240T and Flag-GFP serving as expression control. (c) Densitometric quantification of 3 independent experiments as shown in (b). Compared with the WT variant, hACTN4-M240T is significantly lower expressed (*n* = 3, error bars indicate SD, ∗*P* < 0.05, two-tailed *t* test). GFP, green fluorescent protein; WT, wild-type.

To evaluate the impact of p.M240T exchange within the molecule, we used molecular dynamics analyses based on the solved crystal structure. All algorithms and platforms^12^, ^13^, ^14^, ^15^, ^16^ predicted a strong decrease in stability caused by introduction of the amino acid exchange M240T (Figure 2a). Similar results were obtained for the highly conserved structures of *ACTN1* and *ACTN3*.^25^^,^^26^ In line with these observations, p.M240T showed a reduced expression in human embryonic kidney 293T cells compared with ACTN4-WT (Figure 2b and c).

### ACTN4-M240T Expression Results in Protein Mislocalization and Aggregate Formation

**Figure 3:**
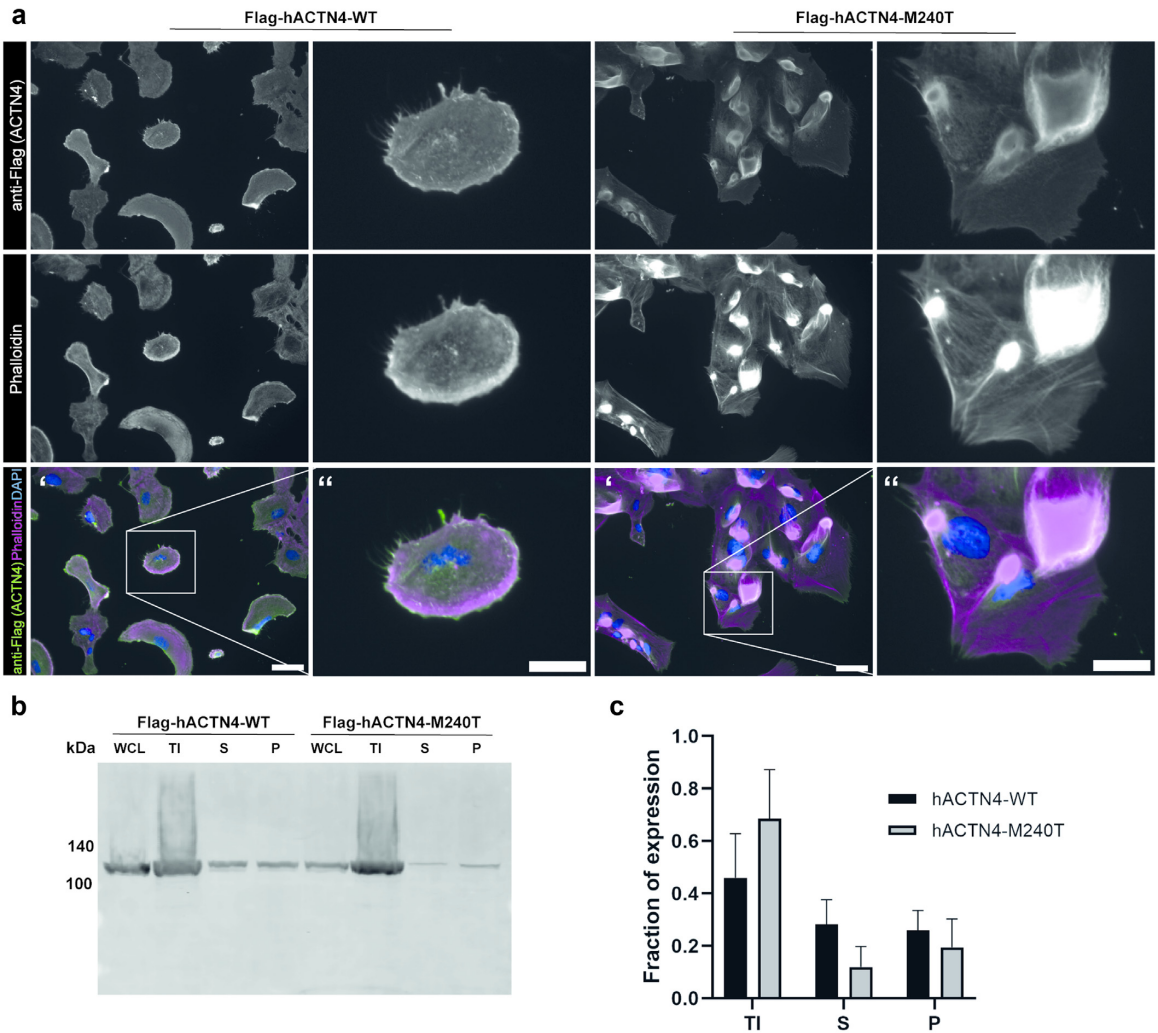
ACTN4-M240T mutation leads to perturbed cellular localization and aggregate formation. (a) Immunofluorescence analysis of podocyte cell lines stably expressing Flag-tagged hACTN4-WT or hACTN4-M240T. Cells were stained with anti-FLAG antibody as well as Phalloidin and 4′,6-diamidino-2-phenylindole to visualize filamentous actin and the nucleus, respectively. In contrast to cells expressing the WT variant, where the protein is located in the cortical region together with F-actin, localization of ACTN4-M240T can be detected more toward the center of the cells, partially associated with large F-actin positive aggregates. Single channels are shown in gray, and scale bar indicates 50 μm in ‘ and 25 μm in ‘‘. (b) Representative western blot analysis of fractionation assays performed in cells transiently expressing either hACTN4-WT or hACTN4-M240T. Whole-cell lysates were subjected to differential centrifugation and the Triton X-100 insoluble as well as the supernatant and pellet derived from the Triton X-100 soluble fraction were analyzed by western blot stained with anti-FLAG antibodies. (c) Densitometric analysis of ACTN4 distribution in TI, S, and P fractions for ACTN4-WT and ACTN4-M240T (*n* = 4). P, pellet; S, supernatant; TI, Triton X-100 insoluble; WCL, whole-cell lysates; WT, wild-type.

To analyze functional consequences of the M240T mutation, we created human podocyte cell lines that either overexpress hACTN4-WT or hACTN4-M240T. In cells expressing hACTN4-WT, the protein could be detected at the cell cortex where it colocalized with F-actin (Figure 3a). In contrast, hACTN4-M240T expression led to severely perturbed localization of the protein. hACTN4-M240T was observed to be located toward the center of the cell forming large aggregates that partially overlapped with F-actin (Figure 3a). Similar observations were made by performing an actin fractionation assay in human embryonic kidney 293T cells. Cell lysates of cells either expressing hACTN4-WT or hACTN4-M240T were subjected to differential centrifugation. The Triton insoluble fraction contains large actin bundles, whereas the Triton soluble fraction, further divided into a pellet and supernatant fraction, contains unbundled filamentous actin and globular actin, respectively. Upon western blot analysis, it was observed that the mutated protein was more abundant in the Triton insoluble fraction (Figure 3b and c), that is, associated with bundled actin.

### Knockdown of Actinin in *Drosophila* Nephrocytes Leads to Reduced ND Length and Filtration Deficits

**Figure 4:**
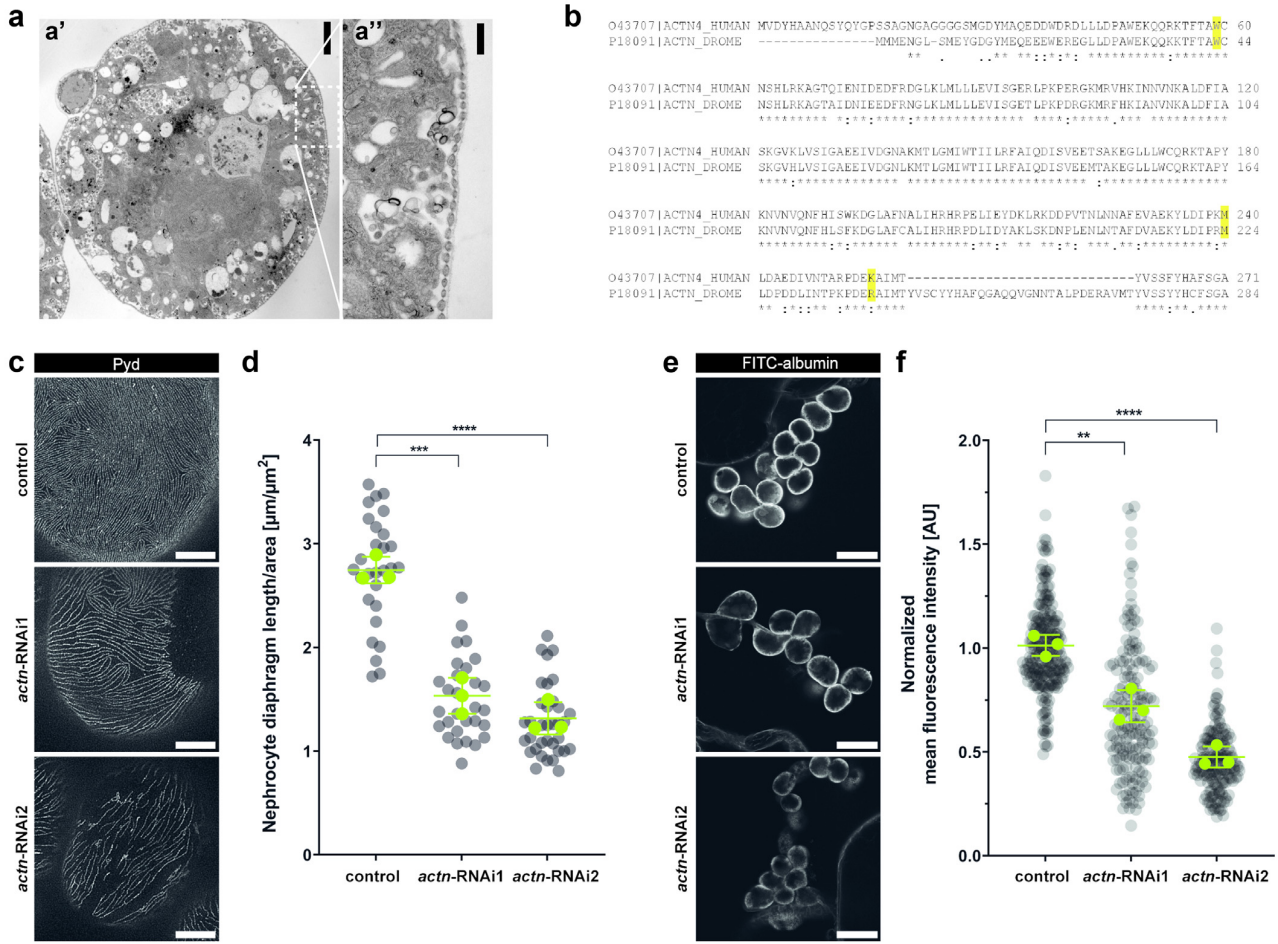
Knockdown of *Drosophila* actinin in nephrocytes results in decreased ND length and reduced filtration function. (a) Electron micrograph depicting a wild-type nephrocyte. Scale bars indicate 5 μm in A’ and 500 nm in A’’ (b) Protein alignment of the N-terminal actin-binding domain of human *ACTN4* and *Drosophila* actinin. Highlighted are amino acids known to be causative of monogenic nephrotic syndrome. Actinin shares 68.83% overall identity with *ACTN4*, the actin-binding domain of the 2 proteins shares 78.8% identity. (c,d) Representative micrographs of nephrocytes stained with (c) anti-Pyd and (d) quantification of the ND length. Nephrocytes derived from either control larvae, or larvae with nephrocyte-specific knockdown of actinin (ACTN), by using 2 different UAS-RNAi-lines (*actn*-RNAi1 and *actn*-RNAi2). Compared with control nephrocytes, the ND length of actn-depleted nephrocytes is significantly reduced (gray dots show all nephrocytes measured, green dots represent means of *n* = 3 independent experiments performed in 3 experimental crossings, error bars indicate SD, ∗∗*P* < 0.01, ∗∗∗*P* < 0.001, one-way ANOVA with Tukey’s *post hoc* test). (e,f) Representative micrographs of nephrocytes subjected to (e) FITC-albumin tracer and (f) quantification of fluorescence intensity as a measure of uptake capacity. Control and *ACTN* knockdown nephrocytes were incubated in 0.2 mg/ml FITC-albumin solution for 30 seconds, and fluorescence intensity was quantified using Fiji. The data are presented as normalized to control levels. Both, *actn-*RNAi1 and *actn-*RNAi2 nephrocytes show a significantly reduced capacity of FITC-albumin uptake with respect to control nephrocytes, indicating severe filtration defects (gray dots show all nephrocytes measured, green dots indicate means of *n* = 3 independent experiments performed in 3 experimental crossings, error bars indicate SD, ∗∗*P* < 0.01, ∗∗∗∗*P* < 0.0001, one-way ANOVA with Tukey’s *post hoc* test). Scale bars indicate 5 μm in (c) and 25 μm in (f). FITC, fluorescein isothiocyanate; ND, nephrocyte diaphragm; Pyd, polychaetoid; RNAi, RNA interference.

Next, we made use of the model organism *Drosophila melanogaster* and analyzed the impact of hACTN4-M240T on nephrocyte morphology and function *in vivo*. Nephrocytes are podocyte-like cells in the fly that built an ND (Figure 4a) via nephrin and NEPH1 homologous proteins Sticks and Stones and Dumbfounded, respectively, and they are responsible for clearing the hemolymph, the fly’s blood equivalent, by an interplay of filtration and endocytosis.^27^^,^^28^ To express human variants of *ACTN4* in a knockdown background, we first aimed to characterize the nephrocyte-specific RNAi-mediated knockdown of endogenous actinin (*ACTN*), the sole homolog of alpha-actinin 1 to 4 proteins in the fly, using the GAL4-UAS-System.^29^ The Actin protein shows an amino acid identity of about 68% with human *ACTN4*, whereby the actin-binding domain in general as well as also single critical amino acids known to be affected in patients with glomerular disease, are especially conserved (Figure 4b). For nephrocyte-specific expression of target genes, the Sticks and Stones-GAL4 driver line^17^ was used. To exclude off-target effects, 2 different *actn*-RNAi lines (*actn*-RNAi1 and *actn*-RNAi2, respectively) were used to achieve nephrocyte-specific depletion of *actn* levels. Overexpression of a short hairpin directed against green fluorescent protein served as control.

To study morphologic consequences of actn depletion in nephrocytes, we first performed immunofluorescent stainings with an antibody directed against polychaetoid, the homolog of ZO-1, which localizes to the ND. Visualizing the cells’ surface by superresolution microscopy, a fingerprint-like pattern of NDs can be detected, which can be quantified as a representation of the ND length (Figure 4b and c) using a previously published ImageJ/Fiji macro.^21^ Knockdown of *actn* resulted in a loosened ND pattern with both RNAi-lines. Quantification showed that the reduction in ND length is comparable in both lines (Figure 4c and d). To analyze nephrocyte function, we performed a tracer uptake assay^19^ and assessed the nephrocytes’ capacity of taking up FITC-labeled albumin. As described before,^30^^,^^31^ knockdown of *actn* led to a severe functional phenotype, as uptake capacity was significantly decreased in both knockdown lines with respect to control nephrocytes (Figure 4e and f).

### hACTN4-M240T Is Not Able to Rescue the *actn* Knockdown Phenotype in *Drosophila*

To elucidate whether hACTN4-M240T is a disease-causing variant of *ACTN4*, we studied its potential to rescue the above-described *actn* knockdown–associated phenotypes in comparison to hACTN4-WT. We also included hACTN4-W59R and hACTN4-K255E in our studies, 2 well-characterized pathogenic *ACTN4* variants. We expressed the individual HA-tagged hACTN4-variants in the background of *actn*-RNAi2, owing to its more pronounced and robust phenotype regarding both morphology and filtration. The newly generated fly lines, which were holding both constructs, UAS-*actn*-RNAi2 and UAS-HA-hACTN4-variant, were validated on the level of protein expression by detection of the protein’s HA-tag in immunofluorescence stainings (Supplementary Figure S1).

**Figure 5:**
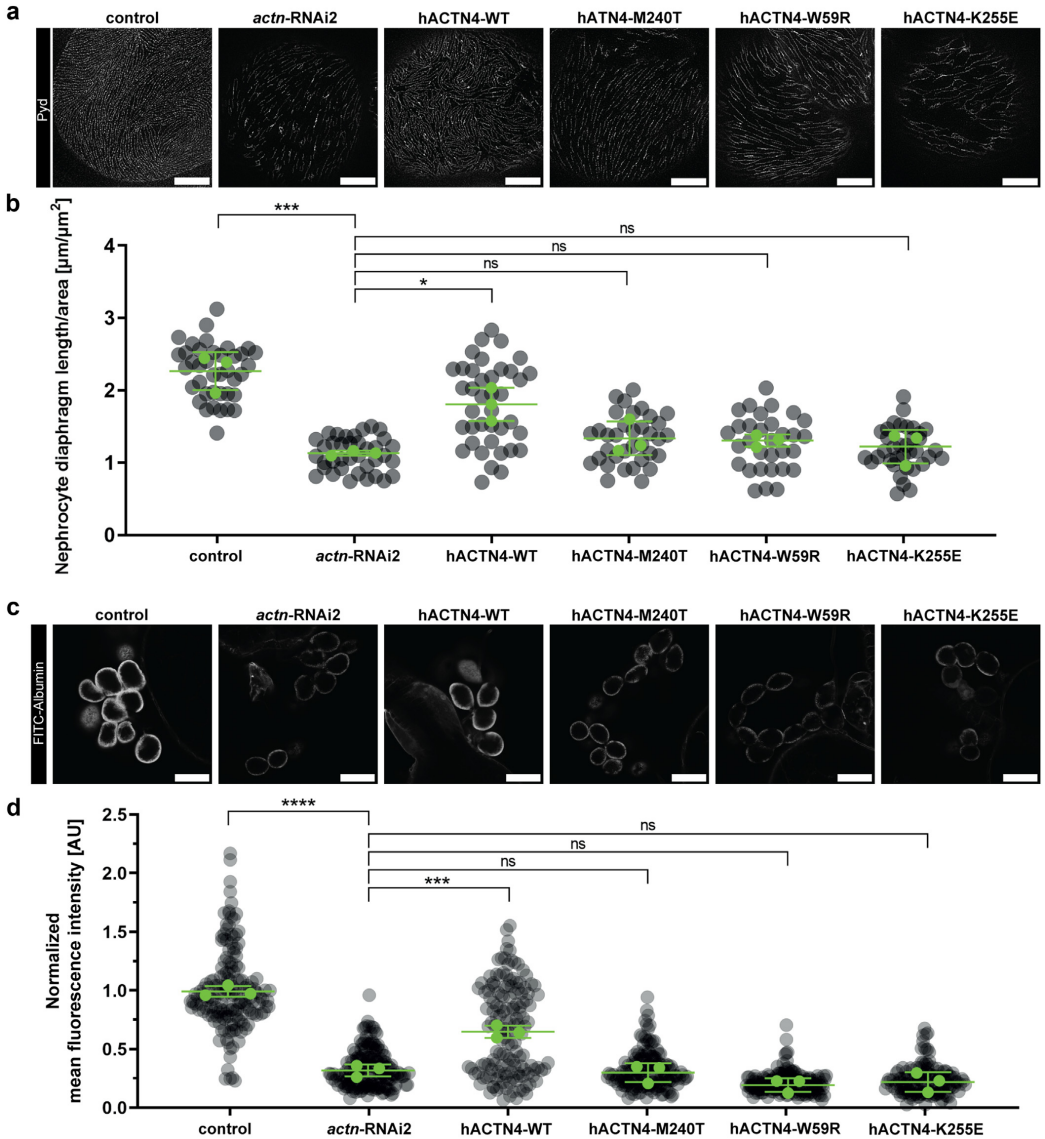
hACTN4-M240T reexpression does not ameliorate actinin knockdown–associated phenotypes. (a,b) Representative micrographs of nephrocytes stained with (a) anti-Pyd and (b) quantification of the ND length. Nephrocytes derived from either control larvae, larvae with nephrocyte-specific knockdown of *actinin* (*actn-*RNAi2) as well as larvae reexpressing the indicated hACTN4-variant in the knockdown background. hACTN4-WT is able to partially rescue the actinin knockdown–associated reduction in ND length, whereas reexpression of hACTN-M240T does not lead to increased ND length. This is also true for FSGS-associated mutations FSGS-W59R and FSGS-K255E (gray dots indicate all nephrocytes measured, green dots show means of *n* = 3 independent experiments performed in 3 experimental crossings, error bars indicate SD, ∗∗∗*P* < 0.001, ∗*P* < 0.05, one-way analysis of variance with Tukey’s *post hoc* test). (c,d) Representative micrographs of nephrocytes subjected to (c) FITC-albumin tracer and (d) quantification of fluorescence intensity as a measure of uptake capacity. Nephrocytes were incubated in 0.2 mg/ml FITC-albumin solution for 30 seconds, and fluorescence intensity was quantified using Fiji. The data are presented as normalized to control levels. Reexpression of hACTN4-WT also leads to a significant increase in tracer uptake capacity, compared with hACTN4-M240T, hACTN4-W59R, and hACTN4-K255E, where a rescue of actinin knockdown–associated reduction of tracer uptake cannot be observed (gray dots indicate all nephrocytes measured, green dots show means of *n* = 3 independent experiments performed in 3 experimental crossings, error bars indicate SD, ∗∗∗*P* < 0.001, ∗∗∗∗*P* < 0.0001, one-way analysis of variance with Tukey’s *post hoc* test). Scale bars indicate 5 μm in (a) and 25 μm in (c). FITC, fluorescein isothiocyanate; ND, nephrocyte diaphragm; Pyd, polychaetoid; WT, wild-type.

When expressing hACTN4-WT in the knockdown background, we could observe a significant rescue capacity of ND length (Figure 5a and b) and of filtration capacity (Figure 5c and d). hACTN4-M240T, however, was not able to ameliorate either the morphologic or the functional phenotype, which was also observed when expressing hACTN-W59R and hACTN-K255E (Figure 5).

## Discussion

In childhood and adolescence, genetic causes are among the most common origins of chronic kidney disease. Even in adults, a significant number of patients carry a known genetic defect that contributes to the development of ESRD.^32^ INF2, TRPC6, and *ACTN4* are the prime examples of autosomal-dominant forms of SRNS, whereas most of the mutations leading to ESRD are inherited in an autosomal recessive manner and often occur as compound heterozygous alleles.^9^^,^^24^^,^^33^ To date, 20 different mutations have been reported for *ACTN4*-associated glomerular disease, most of them (nonsynonymous) missense mutations (HGMD professional 2022.2).

Within the CRU329, we identified disease-causing mutations in *ACTN4* in 3 patients (Supplementary Table S1). One of these mutations was already reported in 3 other patients (p.F153L). A second identified mutation (p.G195D) was analyzed *in vitro* and reported in 2016.^10^ Here, we report on the analysis of the mutation identified in the third patient who developed proteinuria at the age of 4 years and responded temporarily to ciclosporin A. Genetic workup revealed a *de novo* ACTN4 p.M240T single-nucleotide polymorphism. To test the pathogenicity of ACTN4 p.M240T, we established a time-efficient experimental pipeline using *in silico*, *in vitro*, and *in vivo* experiments. ACTN4 p.M240T was found to be less stable and mislocalized, resulting in aggregates disturbing the actin-based cytoskeleton. *In vivo* experiments using the *Drosophila* nephrocyte model confirmed the pathogenicity of hACTN4-M240T and thereby establish the diagnosis for the described patient.

Mutations in *ACTN4* are inherited in an autosomal-dominant fashion and mostly lead to FSGS and ESRD in adult patients,^9^ although several reports including this manuscript describe patients that develop severe kidney disease at a rather young age.^10^^,^^24^ The responsible mutations are located in 1 of the 2 calponin-homology-domains constituting the N-terminal actin-binding domain. Among other scaffolding proteins, *ACTN4* has been shown to colocalize with nephrin at the podocytes’ slit diaphragm.^34^^,^^35^ As part of this group of slit diaphragm complex proteins, it is involved in anchoring the actin cytoskeleton to junctional proteins and the glomerular basement membrane.^35^ Ultimately, *ACTN4* is implicated in several cellular functions and, in particular, to establish and maintain the podocyte’s sophisticated 3D structure, adhesion to the glomerular basement membrane, and signal transduction processes.^36^, ^37^, ^38^ The biological consequences of *ACTN4* mutations and the mechanism by which they lead to disease are not fully understood. It is postulated that a conformational change occurs when *ACTN4*-mutants bind to F-actin, which results in an altered binding affinity for mutant proteins as compared with WT *ACTN4*.^39^ The dissociation rate of *ACTN4*-K255E from F-actin is much slower as compared with the WT protein^40^ and also promotes the formation of F-actin aggregates,^39^ a finding that is also observed with further *ACTN4* mutations including this study.^10^ One might speculate whether the disease is driven by the loss of *ACTN4* function itself or owing to its mislocalization or both. Several other *ACTN4* mutants have been described to be less stable *in vitro* and to be more rapidly degraded whereby protein synthesis remains stable^41^ or to possess a higher affinity for F-actin.^10^^,^^23^^,^^24^^,^^41^ Moreover, *ACTN4* knockout mice develop profound proteinuria and FSGS at the age of 10 weeks and show a decreased number of glomerular podocytes.^36^^,^^42^ Lower expression levels of *ACTN4* were also reported in patients with glomerulopathies including FSGS.^43^

We used the genetic toolbox of *Drosophila melanogaster* to study the impact of *ACTN4*-M240T on nephrocyte morphology and function. The *Drosophila* system has several advantages over higher organisms such as mouse models. In addition to simple and cheap husbandry, the fruit fly stands out with its diverse methods of genetic manipulation.^44^^,^^45^ Numerous commercially available fly lines, including mutant and RNAi libraries and custom-made stocks for the expression of, for example, human transgenes make it possible to study a gene of interest and its genetic interactors in a shorter time frame. It is noteworthy, that although it is an invertebrate system, *Drosophila* shares a high genomic, molecular, and structural conservation with mammals, which is why its use in biomedical research has been and still is increasing.^46^, ^47^, ^48^, ^49^, ^50^, ^51^, ^52^ The rediscovery of nephrocytes and their structural and functional similarity to mammalian podocytes^27^^,^^28^ broadened that scope to glomerular kidney diseases, emphasizing the feasibility of the *Drosophila* nephrocyte system not only to address basic podocyte research but also to accompany diagnostics of glomerular diseases in the future.

The presented case underlines several important aspects regarding diagnostics and treatment in patients with FSGS. First, the trigger leading to the FSGS has to be identified. An in-depth workup is necessary and genetic testing is a central piece in diagnostics, especially when no other overt cause is found. Second, apart from the growing number of known genes and mutations contributing to SRNS and FSGS, sequencing approaches facilitate the establishment of a potential genetic diagnosis.^53^ In this context, it is necessary to keep in mind that there is a substantial rate of polymorphisms or rare benign variants that are not disease-causing.^54^^,^^55^ Therefore, further workup is necessary to provide evidence of whether a novel discovered variant is indeed pathogenic. Besides *in silico* predictions and *in vitro* cell culture experiments, the *Drosophila* nephrocyte is an attractive and feasible system to add *in vivo* data to these critical analyses. This is not just necessary to ensure the diagnosis in the individual patient, but it also helps to guide therapy decisions (e.g., avoiding medications with potentially adverse side effects that are unlikely to be effective in certain conditions) and to counsel the patient and the family regarding future treatment options, such as whether a living-donor kidney transplantation is suggested and possible. Third, the presented case confirms and adds to the growing body of literature that indicates that in cases of FSGS attributed to a genetic cause, immunosuppressive treatment strategies such as ciclosporin A might result at least in a partial response and can delay the time until a kidney replacement therapy or transplantation is necessary.^7^^,^^56^ This positive effect might be attributed to direct effects of certain drugs on podocyte structures such as the cytoskeleton.^57^ Furthermore, proteinuria itself triggers an immune response that accelerates the glomerular injury. This can be controlled—at least to some extent—by immunosuppressive drugs.^58^

The growing complexity in genetics, clinical presentation, molecular characterization of the affected proteins, and individual treatment options of these patients also emphasizes the need for inclusion of patients in registries and databases.^8^^,^^59^^,^^60^ The characterization of unclear variants is key to optimizing patient care and guidance, especially when the clinical presentation and course differ from the published literature. Bioinformatic prediction tools such as the ClinPred score^61^ are helpful to estimate the potential pathogenic impact of novel sequence variants, but formal proof of deleterious effects and altered function should be aimed for in cases where far-reaching clinical decisions such as preemptive living-donor kidney transplantation depend on the genotype data. Of note, the presented cases underline that *ACTN4* mutations should also be considered in young (sporadic) patients with FSGS.

In conclusion, we present a feasible combined way to evaluate the pathogenic potential of novel mutations in podocyte genes. This might be a blueprint for a pipeline analyzing unclear genetic variants in patients with podocyte diseases such as FSGS.

## Disclosure

All the authors declared no competing interests.

## Supplementary Material

**Supplementary Methods**.

**Supplementary References.**

**Figure S1.** Validation of protein expression of hACTN4-variants in *Drosophila* nephrocytes.

**Table S1.** ACNT4 *de novo* variants identified in pediatric SRNS/FSGS cases.
