# Supplementary material for: Modeling of ACTN4-Based Podocytopathy Using Drosophila Nephrocytes
Source: Kidney Int Rep. 2022 Oct 31;8(2):317–29. doi: 10.1016/j.ekir.2022.10.024 (PMC9939316; doi:10.1016/j.ekir.2022.10.024)
Supplement: Supplementary File (PDF) [file mmc1.pdf]

Supplementary Material

Supplementary Methods:

Molecular cloning

Human ACTN4 wildtype plasmids were already available from [1]. Site-directed mutagenesis to clone hACTN4-W59R, -M240T, and -K255E was performed according to standard protocols using hACTN4-WT in a modified pENT1A vector (Invitrogen). For transient transfection of HEK293T cells, the constructs were subcloned into a modified pcDNA6 vector (Invitrogen) containing an N-terminal Flag-tag. Generation of human podocyte cell lines, which stably express Flag-tagged hACTN4-WT or ACTN4-M40T, or transgenic *Drosophila* fly lines expressing HA-tagged hACTN4 variants under UAS control was achieved by recombining the constructs into pLenti6.3 (Invitrogen) or pTHW (Drosophila Genomics Resource Centre), respectively, using standard GATEWAY technology protocols. All plasmids were verified by sequencing.

Cell culture

HEK293T cells were kept in Dulbecco's modified Eagle's medium supplemented with 10 % fetal bovine serum (FBS). Transient transfection of the cells was achieved using standard calcium-phosphate transfection protocols, adding pcDNA6-GFP as a transfection control. HEK293T cells were also utilized for the production of lentivirus using the pLenti6.3 system. Change to regular podocyte growth medium with 20 mM HEPES was done after 8 hours. Virus containing medium was finally collected after 72 hours, filtered, and stored at 4 °C. For stable expression of human ACTN4 variants in podocyte cell culture, a previously described immortalized human podocyte cell line [2] was used. Cells were cultivated in RPMI 1640 medium supplemented with 10 % FBS as well as insulin-transferrin supplement. They were transduced with lentivirus for 24 hours adding polybrene under growth conditions, before performing medium changes every 24 hours. For selection purposes, blasticidin (1 µg/ml) was added to the medium 48 hours after transduction. Cells were used for further experiments after 96 hours.

Western Blot analysis

For western blot analysis, equal amounts of protein were separated by SDS-PAGE and blotted onto a PVDF membrane using semi-dry transfer methods. After blocking in 5 % BSA for 30 minutes and three washing steps in 1x PBS, the membranes were incubated in primary

antibodies in PBS-T (0.1 % Tween in 1x PBS) overnight at 4 °C. The following primary antibodies were used: Mouse anti-Flag (Sigma F1804, 1:10000) and rabbit anti-pan-actin (Cell Signaling #8456). After three washing steps, membranes were incubated in secondary antibodies coupled to horseradish peroxidase (goat anti-mouse, Jackson #115-035-003, goat anti-rabbit, Jackson #111-035-003, 1:15000) or fluorescent secondary antibodies (IRDye 800CW Goat anti-Mouse, LI-COR #926-32210), followed by three further washing steps. Visualization was performed using the FUSION or LI-COR detection systems, respectively.

Immunofluorescence of cultured cells

After splitting the human podocyte cell line onto coverslips and growth to a confluency of about 60 % (24 hours), cells were washed in PBS and fixed in 4 % PFA for 15 minutes at room temperature. After several washing steps and blocking in 5 % normal donkey serum in PBS containing 0.1 % Triton-X for 1 hour, the samples were incubated in primary antibody (mouse anti-Flag, Sigma F1804, 1:1000) overnight at 4 °C. Incubation with secondary antibody (goat anti-mouse 488, Jackson #115-545-003, 1:500) and Phalloidin-Alexa647 (Dyomics, 647P1-33, 1:250) was performed at room temperature for 1 hour. The coverslips were finally mounted in ProLong Diamond + DAPI (Thermofisher, P36971).

Supplementary Figure S1:

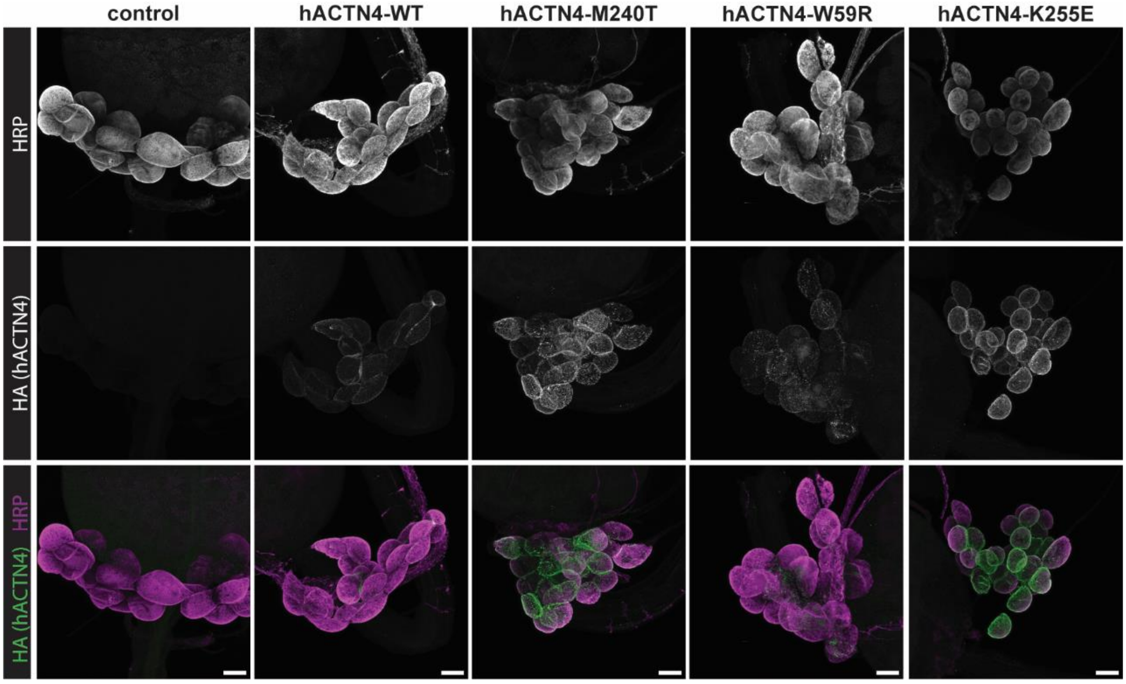

Supplementary Figure S1: Validation of protein expression of hACTN4-variants in *Drosophila* nephrocytes. Immunofluorescence analysis of nephrocytes expressing HA-tagged hACTN4-variants under UAS-control. Nephrocytes were stained with anti-HRP (nephrocyte membrane) and anti-HA (detecting HA-tag of hACTN4-variants). Scale bar indicates 25 µm.

Supplementary Table S1:

**Patient Number**	**1**	**2**	**3**
**HGVS cDNA**	c.457T>C	c.584G>A	c.719T>C
**HGVS protein**	p.F153L	p.G195D	p.M240T
**Mutation type**	missense	missense	missense
**ACMG class**	5	4	4
**Status**	reported thrice	reported once	novel<br>this study
**Reference**<br>**ClinVar**<br>**ClinPred prediction**	PMID: 23014460 [3]<br>NA<br>0.997	PMID: 26740551 [1]<br>1 submission<br>0.999	this study<br>2 submissions<br>0.998
**AF gnomAD**	not found	not found	not found
**Patient course**	Clinical presentation at age 17 with SRNS, KB: FSGS.<br>PR with cyclosporine A (reduction of proteinuria from 15g/d to 6-8g/d), moderate decline in kidney function	Clinical presentation with NS and ESRD at age 13,<br>initiation of PD,<br>L-KTX one year later	Clinical presentation at age 4 with proteinuria, first KB: MCD; persisting proteinuria, second KB: FSGS, PR with cyclosporine A; progression to ESRD and preemptive L-KTX at 9 years of age

Supplemental Table S1: ***ACNT4 de novo*** **variants identified in pediatric SRNS /FSGS cases.**

Abbreviations: NS, nephrotic syndrome, PD, peritoneal dialysis; L-KTX, living donor kidney transplantation; PR, partial remission; KB, kidney biopsy; MCD, minimal change disease; FSGS, focal segmental glomerulosclerosis

Supplementary References

1. Bartram MP, Habbig S, Pahmeyer C, et al. Three-layered proteomic characterization of a novel ACTN4 mutation unravels its pathogenic potential in FSGS. *Hum Mol Genet*. 2016;25(6):1152-1164.
2. Saleem MA, O'Hare MJ, Reiser J, et al. A conditionally immortalized human podocyte cell line demonstrating nephrin and podocin expression. *J Am Soc Nephrol*. 2002;13(3):630-638.
3. Barua M, Brown EJ, Charoonratana VT, Genovese G, Sun H, Pollak MR. Mutations in the INF2 gene account for a significant proportion of familial but not sporadic focal and segmental glomerulosclerosis. *Kidney Int*. 2013;83(2):316-322.
